# Inhaled nitric oxide use facilitated VA-ECMO weaning during ECPELLA support in a patient with a precapillary pulmonary vascular phenotype: a case report

**DOI:** 10.1093/ehjcr/ytag788

**Published:** 2026-10-22

**Authors:** Motomaro Tanaka, Takashi Unoki, Hiroki Matsushita, Keita Saku, Junjiro Koyama

**Affiliations:** Division of Cardiology, Saiseikai Kumamoto Hospital, 5-3-1 Chikami, Minami-ku, Kumamoto 861-4193, Japan; Division of Cardiology, Saiseikai Kumamoto Hospital, 5-3-1 Chikami, Minami-ku, Kumamoto 861-4193, Japan; Department of Cardiovascular Dynamics, National Cerebral and Cardiovascular Center, 6-1 Kishibe-Shimmachi, Suita, Osaka 564-8565, Japan; Department of Cardiovascular Dynamics, National Cerebral and Cardiovascular Center, 6-1 Kishibe-Shimmachi, Suita, Osaka 564-8565, Japan; Department of Cardiovascular Dynamics, National Cerebral and Cardiovascular Center, 6-1 Kishibe-Shimmachi, Suita, Osaka 564-8565, Japan; Division of Cardiology, Saiseikai Kumamoto Hospital, 5-3-1 Chikami, Minami-ku, Kumamoto 861-4193, Japan

**Keywords:** Inhaled nitric oxide, VA-ECMO, Impella, ECPELLA, Pulmonary vascular resistance, Case report

## Abstract

**Background:**

Combined support with veno-arterial extracorporeal membrane oxygenation (VA-ECMO) and a micro-axial flow pump (mAFP), termed ECPELLA, provides systemic circulatory support with left ventricular (LV) unloading in refractory cardiogenic shock. VA-ECMO weaning may nevertheless be difficult when pulmonary vascular afterload is increased despite adequate LV unloading. Inhaled nitric oxide (iNO) selectively reduces pulmonary vascular resistance (PVR), but its role in this setting remains unclear.

**Case summary:**

An 83-year-old woman with non-ST-segment elevation acute coronary syndrome underwent percutaneous coronary intervention. Persistent circulatory collapse required mAFP support followed by VA-ECMO. Despite adequate LV unloading, pulmonary artery catheterization showed low pulmonary artery catheter-derived cardiac output (PAC-CO), low pulmonary capillary wedge pressure, and high calculated PVR, suggesting increased pulmonary vascular afterload rather than residual LV congestion. VA-ECMO flow reduction caused hypotension. After administration of iNO at 40 ppm, PAC-CO increased from 1.2 to 1.5 L/min and calculated PVR decreased from 11.7 to 8.0 Wood units while mechanical circulatory support settings and ventilator settings remained unchanged. VA-ECMO was removed on Day 2 and the mAFP on Day 3. The patient nevertheless died on Day 4 from complications of severe lower-limb ischaemia.

**Discussion:**

A relative increase in PVR may hinder VA-ECMO weaning during ECPELLA support. iNO may facilitate device weaning in selected patients; however, a temporal association in a single case does not establish causality, clinical recovery, or a survival benefit.

Learning points

- During ECPELLA support, assess right ventricular function and distinguish pulmonary vascular from left atrial contributions to right ventricular afterload.
- Inhaled nitric oxide may improve right ventricular output and left ventricular filling, facilitating VA-ECMO weaning.

## Introduction

Veno-arterial extracorporeal membrane oxygenation (VA-ECMO) provides rapid systemic circulatory support but increases left ventricular (LV) afterload, potentially worsening pulmonary congestion and delaying recovery.^1,2^ A micro-axial flow pump (mAFP) unloads the LV, and combined VA-ECMO and mAFP support (ECPELLA) is used for severe cardiogenic shock.^3,4^ mAFP use during percutaneous coronary intervention has increased.^5^ Right ventricular (RV) dysfunction and pulmonary haemodynamics are prognostic in cardiogenic shock and VA-ECMO support.^6–8^ Despite LV unloading, increased pulmonary vascular afterload may limit native cardiac output (CO) and hinder VA-ECMO weaning.^9^

Inhaled nitric oxide (iNO) reduces pulmonary vascular resistance (PVR) without systemic hypotension and is widely used for acute RV failure.^10^ Although case reports describe iNO use during ECPELLA support for fulminant myocarditis or acute myocardial infarction with RV dysfunction,^11,12^ its role when pulmonary vascular afterload limits VA-ECMO weaning remains unclear.

## Summary figure

**Figure:**
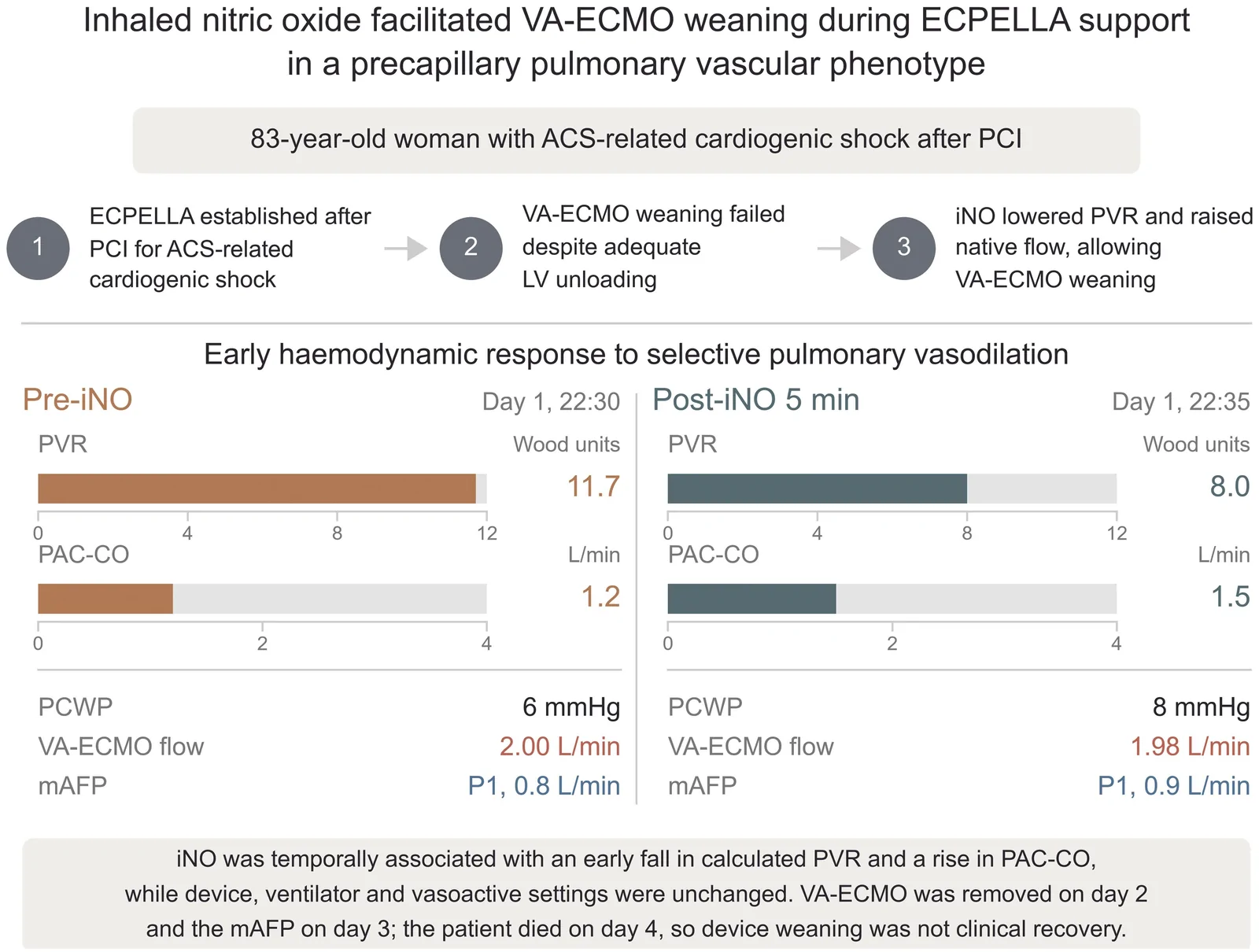


## Case presentation

An 83-year-old woman with multiple cardiovascular comorbidities, including severe peripheral artery disease, presented with progressive chest discomfort, vomiting, impaired consciousness, and recurrent ventricular tachycardia. She had no known chronic lung disease, venous thromboembolism, or connective tissue disease. She was intubated at the referring hospital for worsening haemodynamic instability and transferred to our institution for advanced mechanical circulatory support (MCS).

On arrival, blood pressure was 95/76 mmHg and heart rate 134 b.p.m. with an irregular rhythm while receiving noradrenaline at 0.3 μg/kg/min. She was unconscious and mechanically ventilated. Examination revealed bilateral pulmonary crackles, cold extremities, and livedo reticularis. Electrocardiography and transthoracic echocardiography suggested anterior ischaemia.

Emergent coronary angiography revealed 90% stenosis of the proximal left anterior descending artery, which was treated with drug-eluting stent implantation (*Figure 1*). Despite successful revascularization, systemic hypoperfusion persisted, requiring mAFP insertion. Ongoing circulatory collapse required VA-ECMO, establishing ECPELLA support.

**Figure 1:**
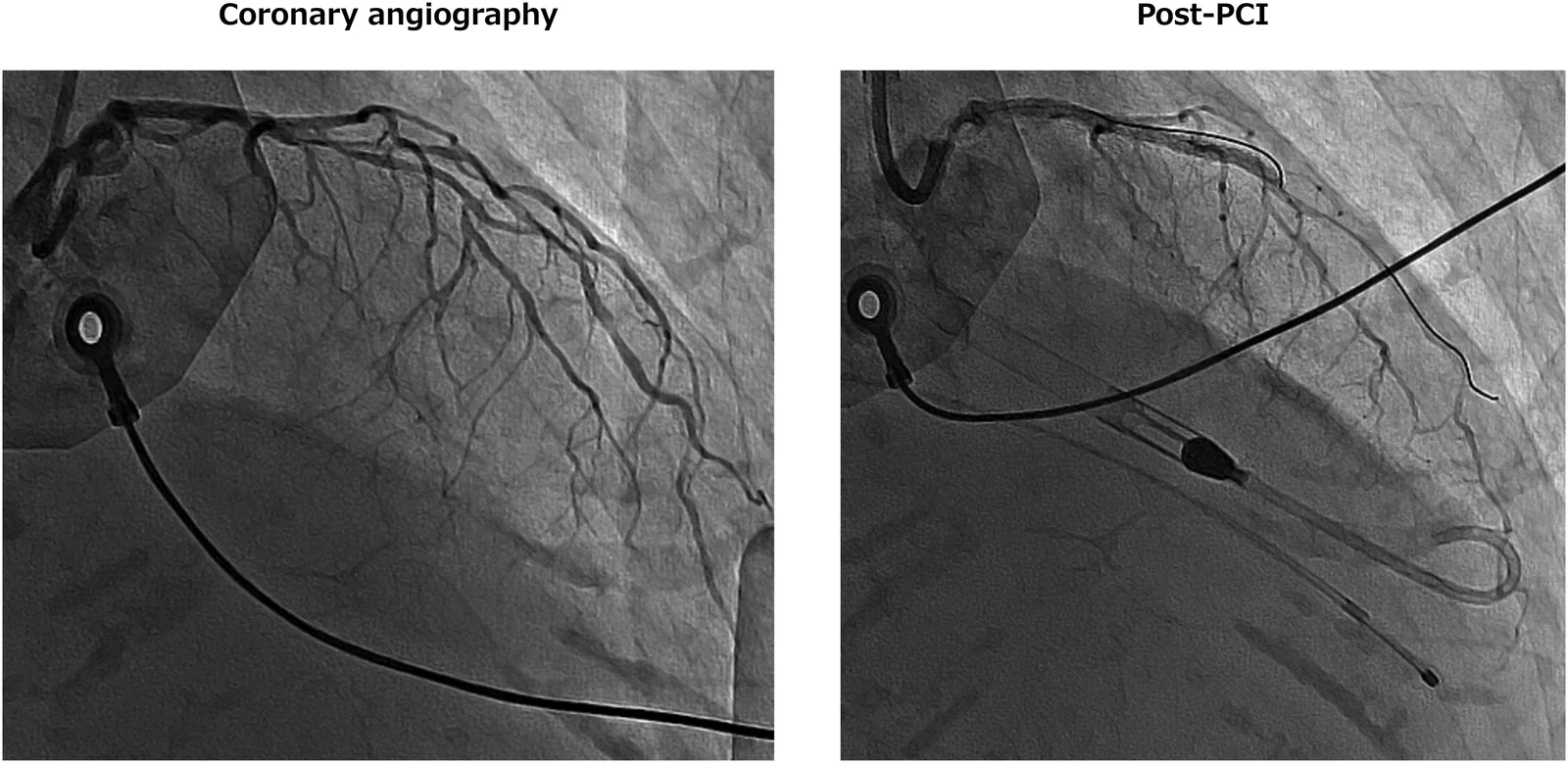
Emergent coronary angiography demonstrated 90% stenosis of the proximal left anterior descending artery. Percutaneous coronary intervention with drug-eluting stent implantation was performed. Because systemic hypoperfusion persisted despite successful revascularization, a micro-axial flow pump was subsequently inserted.

VA-ECMO flow reduction caused hypotension, indicating inadequate native circulatory support. At that time, pulmonary artery catheter-derived CO (PAC-CO) was 1.2 L/min, mean pulmonary arterial pressure (mPAP) 20 mmHg, pulmonary capillary wedge pressure (PCWP) 6 mmHg, calculated PVR 11.7 Wood units, and pulmonary artery pulsatility index (PAPi) 3.00 (*Figure 2* and *Table 1*), suggesting a relative increase in PVR rather than residual LV congestion.

**Figure 2:**
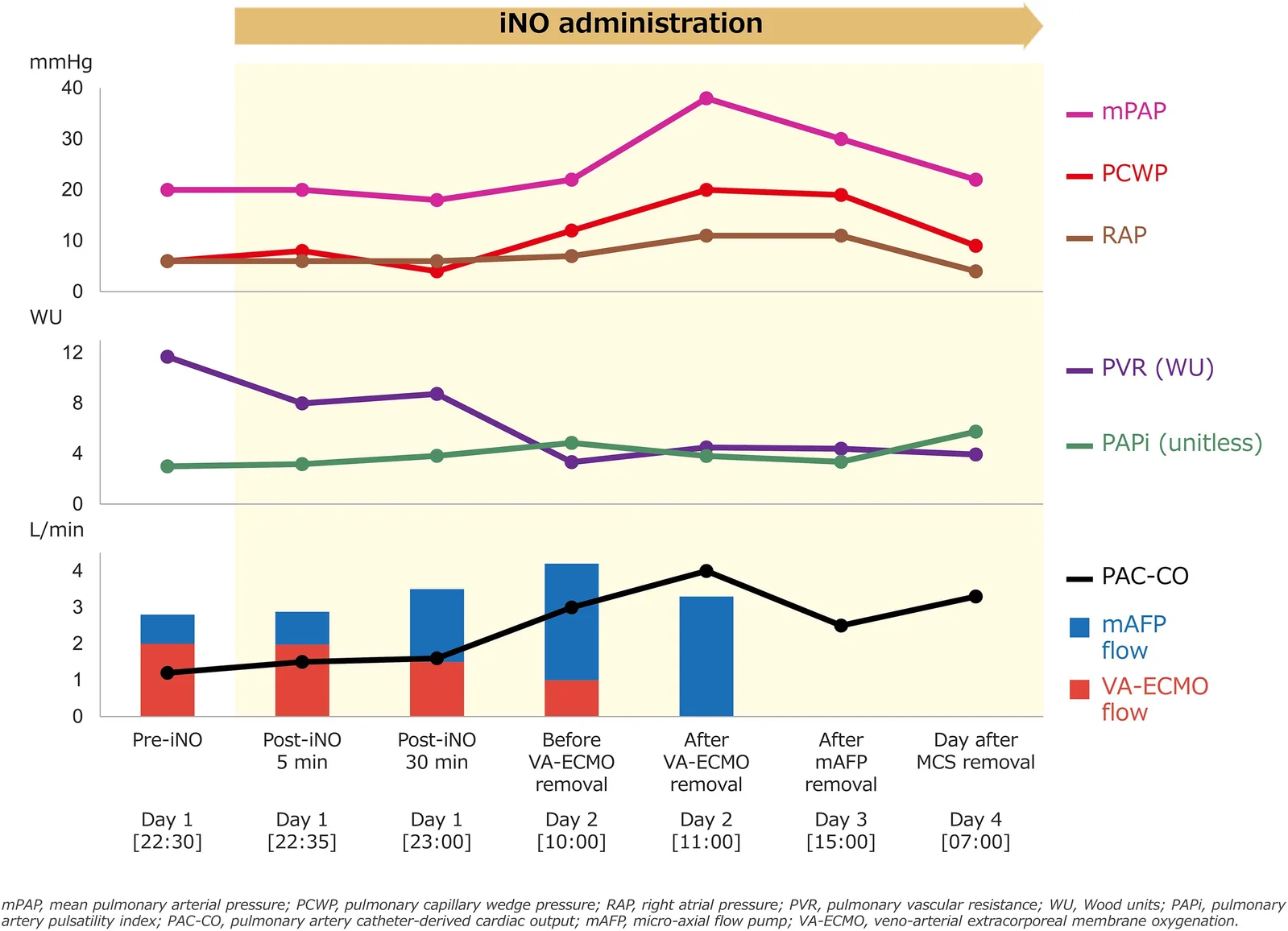
Patient’s haemodynamic data during veno-arterial extracorporeal membrane oxygenation (VA-ECMO) weaning under ECPELLA support (VA-ECMO plus a micro-axial flow pump [mAFP]). Right heart catheterization demonstrated markedly elevated calculated pulmonary vascular resistance (PVR) with low pulmonary capillary wedge pressure and markedly reduced pulmonary artery catheter-derived cardiac output (PAC-CO), with a PAC-CO of 1.2 L/min, a mean pulmonary arterial pressure of 20 mmHg, calculated PVR of 11.7 Wood units, and pulmonary capillary wedge pressure of 6 mmHg. Inhaled nitric oxide (iNO) was therefore initiated at 40 ppm. After iNO initiation, PAC-CO increased from 1.2 to 1.5 L/min and calculated PVR decreased from 11.7 to 8.0 Wood units; VA-ECMO flow reduction was subsequently better tolerated. The time points shown are Day 1 at 22:30 (pre-iNO), Day 1 at 22:35 (5 min after iNO intiation), Day 1 at 23:00 (30 min after iNO initiation), Day 2 at 10:00 (before VA-ECMO removal), Day 2 at 11:00 (after VA-ECMO removal), Day 3 at 15:00 (after mAFP removal), and Day 4 at 07:00 (Day after MCS removal).

**Table 1:** Haemodynamic changes before and after inhaled nitric oxide initiation and during mechanical circulatory support weaning

Parameter	Pre-iNO Day-1 [22:30]	Post-iNO 5 min Day-1 [22:35]	Post-iNO 30 min Day-1 [23:00]	Before VA-ECMO removal Day-2 [10:00]	After VA-ECMO removal Day-2 [11:00]	After mAFP removal Day-3 [15:00]	Day after MCS removal Day-4 [07:00]
sBP, mmHg	60	65	62	88	96	121	98
dBP, mmHg	49	51	56	57	56	53	46
mBP, mmHg	53	55	58	64	66	76	66
HR, beats/min	61	64	63	72	101	64	73
EtCO_2_, mmHg	11	24	28	35	36	36	28
VA-ECMO flow, L/min	2.00	1.98	1.50	1.00	—	—	—
VA-ECMO rotation speed, rpm	1660	1660	1660	1200	—	—	—
mAFP support level	P1	P1	P4	P8	P8	—	—
mAFP flow range, L/min (maximum-minimum)	2.0–0.1	2.1–1.0	2.4–1.8	3.7–2.8	3.6–3.0	—	—
mAFP flow, L/min	0.8	0.9	2.0	3.2	3.3	—	—
PAC-CO, L/min	1.2	1.5	1.6	3.0	4.0	2.5	3.3
SvO_2_, %	80	87	79	85	82	66	64
TSP, L/min	3.20	3.48	3.10	4.00	4.00	2.50	3.30
PCWP, mmHg	6	8	4	12	20	19	9
RAP, mmHg	6	6	6	7	11	11	4
sPAP, mmHg	32	33	34	46	63	59	38
dPAP, mmHg	14	14	11	12	21	19	15
mPAP, mmHg	20	20	18	22	38	30	22
PVR, Wood units	11.70	8.00	8.75	3.33	4.50	4.40	3.94
PAPi	3.00	3.17	3.83	4.86	3.82	3.64	5.75

Abbreviations: dBP, diastolic blood pressure; EtCO_2_, end-tidal carbon dioxide; HR, heart rate; iNO, inhaled nitric oxide; mAFP, micro-axial flow pump; mBP, mean blood pressure; PAC-CO, pulmonary artery catheter-derived cardiac output; PAP, pulmonary artery pressure; PAPi, pulmonary artery pulsatility index; PCWP, pulmonary capillary wedge pressure; PVR, pulmonary vascular resistance; RAP, right atrial pressure; sBP, systolic blood pressure; SvO_2_, mixed venous oxygen saturation; TSP, total systemic perfusion; VA-ECMO, veno-arterial extracorporeal membrane oxygenation.

PVR was calculated as (mPAP − PCWP)/PAC-CO. PAPi was calculated as (sPAP − dPAP)/RAP. TSP was calculated as VA-ECMO flow + PAC-CO.

At our institution, iNO is routinely used during VA-ECMO weaning under a local protocol. With the monitoring system used at our institution, PAC-CO values below 1.0 L/min cannot be quantified reliably; consequently, PVR derived from these values cannot be estimated reliably. Because the haemodynamic response to iNO is difficult to evaluate when pulmonary blood flow is minimal, the protocol initiates iNO once PAC-CO exceeds 1.0 L/min. In this patient, a PCWP of 6 mmHg, PAC-CO of 1.2 L/min, calculated PVR of 11.7 Wood units, and intolerance to VA-ECMO flow reduction despite LV unloading suggested pulmonary vascular afterload mismatch and prompted initiation of iNO at 40 ppm. During the first 5 min, VA-ECMO rotational speed, mAFP support at P1, ventilation mode, fraction of inspired oxygen, positive end-expiratory pressure, respiratory rate, and vasoactive-agent doses remained unchanged; no fluid bolus was administered. PAC-CO increased from 1.2 to 1.5 L/min, and calculated PVR decreased from 11.7 to 8.0 Wood units. PAPi was 3.00 before iNO and 3.17 at 5 min. After the mAFP support level was increased from P1 to P4, PAPi was 3.83 at 30 min. VA-ECMO support was reduced stepwise, with decannulation on Day 2 and mAFP removal on Day 3. No rebound increase in pulmonary arterial pressure or methaemoglobinaemia occurred during iNO tapering. Despite transient circulatory stabilization, lower-extremity ischaemia caused myonephropathic metabolic syndrome, refractory metabolic derangements, and multiorgan failure. The patient died on Day 4.

## Discussion

This case illustrates how a relative increase in PVR may reduce pulmonary venous return and LV preload, hindering VA-ECMO weaning despite LV unloading. Under ECPELLA support, VA-ECMO venous drainage reduces native pulmonary blood flow, and device-dependent loading conditions limit direct application of conventional haemodynamic criteria for pulmonary hypertension. In this patient, however, mPAP was 20 mmHg despite a PCWP of 6 mmHg and a PAC-CO of only 1.2 L/min, yielding a transpulmonary pressure gradient of 14 mmHg and a calculated PVR of 11.7 Wood units. We therefore interpreted the transpulmonary pressure gradient as disproportionately high for the prevailing low native pulmonary flow, in the absence of elevated left-sided filling pressure, consistent with a precapillary pulmonary vascular phenotype within the ECPELLA setting. Reports of iNO use during ECPELLA support remain limited.^9,11,12^

The RV is highly sensitive to afterload, and elevated PVR may reduce RV forward flow despite adequate LV unloading.^6^ Because VA-ECMO weaning requires sufficient native CO as extracorporeal flow decreases,^2,13^ markedly reduced PAC-CO and high calculated PVR despite low PCWP, together with intolerance to flow reduction, suggested RV afterload mismatch as an important contributor to weaning difficulty.

iNO is a selective pulmonary vasodilator that can reduce PVR without causing systemic hypotension.^10^ The initial haemodynamic response occurred while VA-ECMO rotational speed, mAFP support at P1, ventilator settings, and vasoactive-agent doses were unchanged, and no fluid bolus was administered, supporting a temporal association with iNO. After 5 min, VA-ECMO flow and mAFP support were modified; therefore, subsequent haemodynamic improvement cannot be attributed to iNO alone and may reflect both pulmonary vasodilation and optimization of MCS. Stepwise VA-ECMO flow reduction was subsequently tolerated without an increase in PCWP. These observations suggest that iNO may be considered as an adjunct when pulmonary vascular afterload contributes to weaning difficulty. Observational data have also associated iNO use with successful VA-ECMO weaning and improved RV function.^9^

PAPi, calculated as (systolic pulmonary arterial pressure − diastolic pulmonary arterial pressure)/right atrial pressure, is associated with successful VA-ECMO weaning.^7^ However, it is influenced by stroke volume, right atrial pressure, pulmonary arterial capacitance, PVR, and PCWP and does not directly measure RV contractility.^14^ During ECPELLA support, preserved PAPi may therefore coexist with impaired RV forward flow. In this case, PAPi 3.00 required interpretation with PAC-CO, PVR, filling pressures, MCS settings, and the response to VA-ECMO flow reduction.

Patient-specific circulatory simulation provided mechanistic context but not direct pressure-volume measurements. Before iNO, the simulated RV loop was vertically elongated. At 5 min, the RV loop shifted downward and leftward and the LV loop rightward, consistent with decreased RV afterload and increased LV preload (*Figure 3A*). Calculated PVR was 8.0 Wood units at 5 min and 8.75 Wood units at 30 min. From 30 min to immediately before VA-ECMO removal, PVR decreased to 3.33 Wood units and the simulated RV loop widened (*Figure 3B*). Because MCS settings changed, this later change cannot be attributed to iNO alone. These model-derived findings are hypothesis-generating.

**Figure 3:**
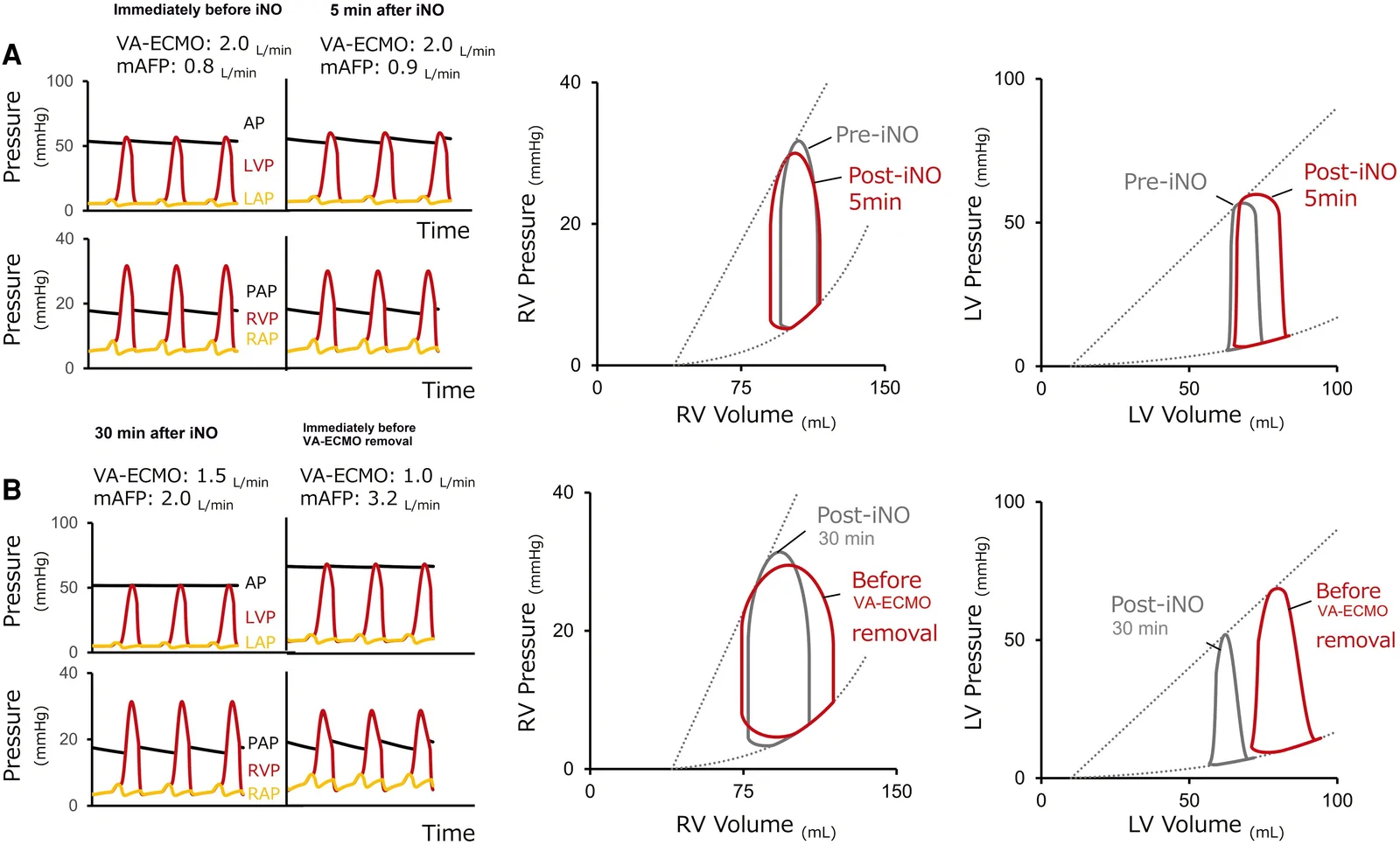
Simulated pressure-volume loops derived from patient-specific haemodynamic data. (*A*) Comparison before iNO and 5 min after iNO initiation, while VA-ECMO rotational speed remained unchanged and the mAFP support level remained at P1. (*B*) Comparison at 30 min after iNO initiation and immediately before VA-ECMO removal, during an interval in which VA-ECMO flow and mAFP support were adjusted. The pressure-volume loops were generated using a patient-specific circulatory simulation model and were not directly measured. The simulation methodology was developed by our group. RV, right ventricular; LV, left ventricular; AP, arterial pressure; LVP, left ventricular pressure; LAP, left atrial pressure; PAP, pulmonary arterial pressure; RVP, right ventricular pressure; RAP, right atrial pressure; mAFP, micro-axial flow pump; iNO, inhaled nitric oxide; P1, performance level 1; VA-ECMO, veno-arterial extracorporeal membrane oxygenation.

This case has several limitations. First, causality cannot be established. Second, VA-ECMO flow reduction and changes in mAFP flow, vasoactive agents, ventilator settings, volume status, and acid-base status may have influenced haemodynamics. Third, during ECPELLA support, PAC-CO was used as an index of native pulmonary flow but may be affected by VA-ECMO flow, mAFP support, and altered loading conditions; PAC-CO and derived PVR values should therefore be interpreted cautiously. Successful VA-ECMO decannulation represented a device-weaning endpoint and should not be interpreted as evidence of clinical recovery or a survival benefit.

Collectively, iNO may be a useful adjunct when a relative increase in pulmonary vascular afterload hinders VA-ECMO weaning during ECPELLA support. Further evidence is needed to identify responsive patients and determine its impact on clinical outcomes.

## Lead author biography


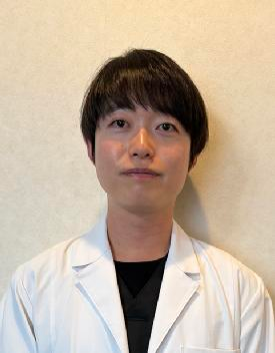


Motomaro Tanaka, MD, is a cardiologist at Saiseikai Kumamoto Hospital, Kumamoto, Japan. His clinical interests include interventional cardiology, mechanical circulatory support, and the management of cardiogenic shock. He is particularly interested in haemodynamic assessment and treatment strategies for critically ill patients requiring veno-arterial extracorporeal membrane oxygenation and Impella support. His research focuses on improving clinical outcomes in patients with severe cardiovascular disease through detailed physiological evaluation and multidisciplinary care.

## Data Availability

All data underlying this article are included within the article.
